# *TMEM151A* variants cause paroxysmal kinesigenic dyskinesia

**DOI:** 10.1038/s41421-021-00322-w

**Published:** 2021-09-13

**Authors:** Hong-Fu Li, Yu-Lan Chen, Ling Zhuang, Dian-Fu Chen, Hua-Zhen Ke, Wen-Jiao Luo, Gong-Lu Liu, Sheng-Nan Wu, Wen-Hao Zhou, Zhi-Qi Xiong, Zhi-Ying Wu

**Affiliations:** 1 Department of Neurology and Research Center of Neurology in Second Affiliated Hospital, Zhejiang University School of Medicine, and Key Laboratory of Medical Neurobiology of Zhejiang Province, Hangzhou, Zhejiang, China; 2 Institute of Neuroscience and State Key Laboratory of Neuroscience, CAS Center for Excellence in Brain Science and Intelligence Technology, Chinese Academy of Sciences, Shanghai, China; 3 University of Chinese Academy of Sciences, Beijing, China; 4 Department of Neurology, Huashan Hospital, Shanghai Medical College, Fudan University, Shanghai, China; 5 Laboratory for Molecular Diagnostics, Shanghai Children’s Hospital, Shanghai Jiao Tong University, Shanghai, China; 6 Department of Neonatology, Children’s Hospital, Fudan University, Shanghai, China

**Keywords:** Endoplasmic reticulum, Rare variants

Dear Editor,

Paroxysmal kinesigenic dyskinesia (PKD) (MIM 128200) is an autosomal dominant movement disorder characterized by involuntary movements which are usually triggered by sudden movements. We previously identified *PRRT2* as the first causative gene of PKD^1^, which was widely verified in different populations^2–4^. It is estimated that *PRRT2* variants account for 77%–93% familial PKD and 21%–45% isolated PKD^5^. A significant percentage of *PRRT2*-negative PKD patients indicate that other genes are implicated in PKD. Here, we performed whole-exome sequencing (WES) in 5 PKD pedigrees and 31 isolated PKD patients without *PRRT2* variants. Moreover, 1000 unrelated healthy individuals of matched ethnicity were included as control subjects. This study was approved by the local Ethics Committee. All participants or their guardians provided written informed consents.

To systematically identify the causative genes for these *PRRT2*-negative PKD patients, we first analyzed three PKD families with multiple patients and unaffected individuals. Based on the autosomal dominant inheritance pattern in these families, we prioritized the heterozygous nonsynonymous variants in coding regions and splicing sites, with a minor allele frequency (MAF) <0.01% in the genome Aggregation Database (gnomAD), and absent in the 1000 Genomes Project (1000G), Exome Sequencing Project v.6500 (ESP6500), Exome Aggregation Consortium (ExAC). Besides, variants should be shared by all affected individuals but not by the unaffected parent of the proband in each family. After filtering, we found 11 candidate genes in Family 1, 29 in Family 2, and 52 in Family 3 (Supplementary Table S1). The comparison of these candidate genes revealed that *TMEM151A* (NM_153266) was the only gene implicated in these families. Three *TMEM151A* variants including c.1275dupG (p.P426Afs*19), c.375 C > A (p.C125X), and c.758 T > C (p.L253P) were confirmed by Sanger sequencing and co-segregation analysis (Fig. 1a). We did not find any *TMEM151A* variant in the other two PKD families. The detailed WES data are shown in Supplementary Table S2. We then screened *TMEM151A* variants in the WES data of 31 isolated PKD patients. We found four truncated variants (c.7 G > T [p.E3X], c.623_624insA [p.L210Afs*136], c.739 G > T [p.E247X], and c.897_912del [p.L300Pfs*118]), three missense variants (c.140 T > C [p.L47P], c.863 T > C [p.F288S], c.889 T > A [p.S297T]), and a non-frameshift deletion (c.142_153del [p.48_51delLTLL]) in 8 index patients (Fig. 1b and Supplementary Table S3).

**Fig. 1:**
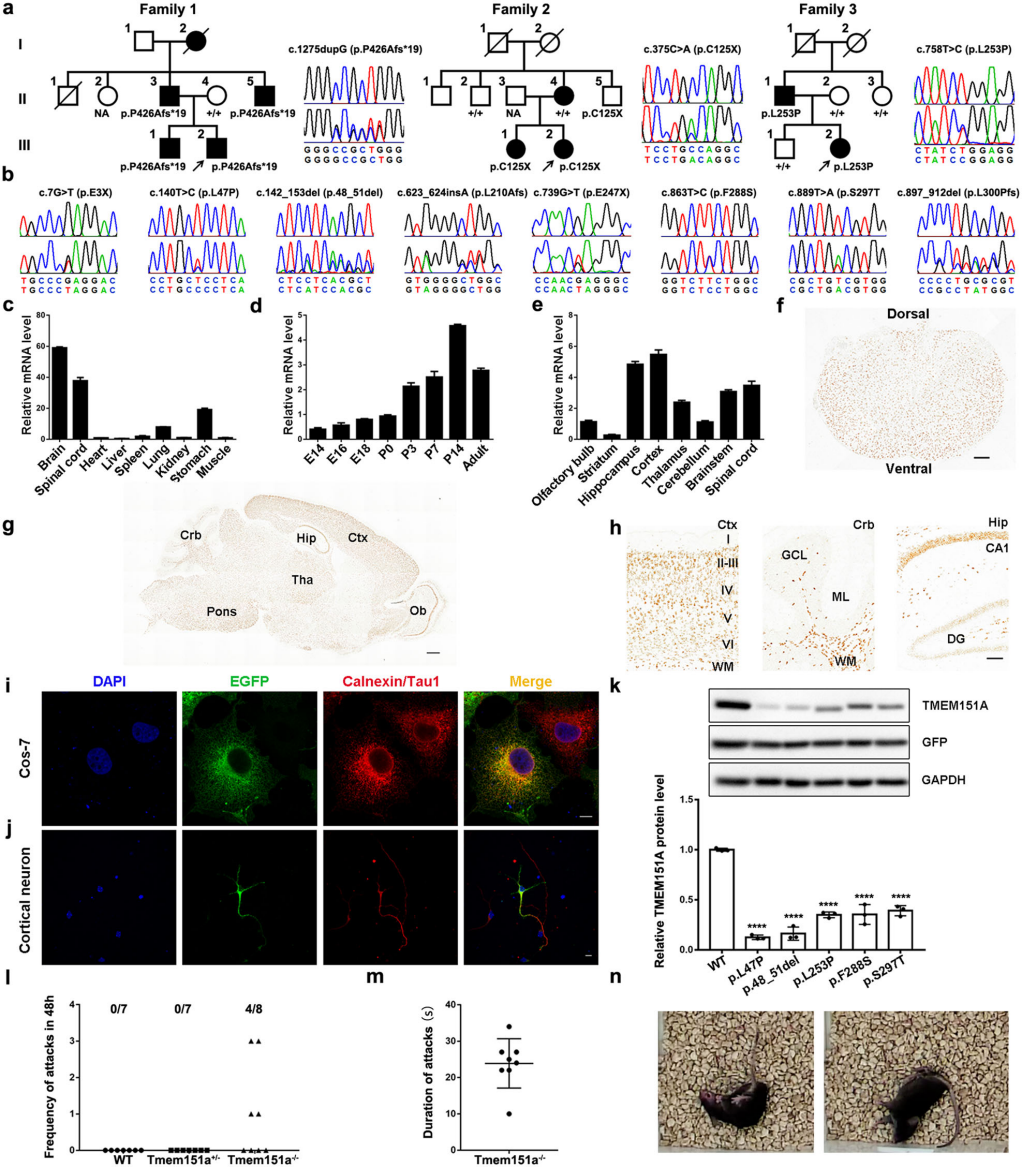
*TMEM151A* variants cause paroxysmal kinesigenic dyskinesia. **a** The pedigree structure and segregation analyses of Families 1–3 and sequencing chromatograms of the identified *TMEM151A* variants. The upper chromatogram represents the normal sequence, and the lower represents the variant. Square: male; circle: female; arrow: index patient; filled symbol: affected; open symbol: unaffected; +: wild-type allele. **b** Sequencing chromatograms of *TMEM151A* variants identified in eight isolated PKD patients. **c** Relative expression level of *Tmem151a* mRNA in various organs of 2-month-old mice. The expression level in the heart was used as a calibration (means ± SEM, *n* = 3). **d** Relative expression level of *Tmem151a* mRNA in the developing mouse brain. The expression level in P0 was used as a calibration (means ± SEM, *n* = 3). **e** Relative expression level of *Tmem151a* mRNA in different regions of the central nervous system. The expression level in the olfactory bulb was used as a calibration (means ± SEM, *n* = 3). **f** In situ hybridization for *Tmem151a* in the P14 mouse spinal cord. Scale bar, 200 µm. **g**, **h** In situ hybridization for *Tmem151a* in the P14 mouse brain. Ctx cortex, Hip hippocampus, Ob olfactory bulb, Crb cerebellum, Tha thalamus, DG dentate gyrus, ML molecular layer, GCL granule cell layer, WM white matter. Roman numerals (I–VI) indicate layers of the cerebral cortex. Scale bar, 500 µm in **g**, 100 µm in **h**. **i**, **j** Fluorescence images of COS-7 cells (**i**) and primary cortical neurons (**j**) transfected with EGFP-tagged Tmem151a and immunostained with antibodies against GFP (green) and either the ER marker calnexin (red) or anti-Tau1 antibodies (axon marker, red). DAPI (blue) was used for nuclear staining. Scale bar, 10 µm. **k** Western blots of protein obtained from HEK293T cells transfected with WT and mutant pIRES2-Flag-TMEM151A plasmids. The anti-Flag antibody was used to detect the TMEM151A protein. The GFP was used to measure the transfection efficiency and served as an internal control. Bar graph shows protein expression level. Data are presented as means ± SD, *n* = 3, ****P < 0.0001. **l** The number of dyskinesia attacks in WT, heterozygous *Tmem151a*^*+/−*^, and homozygous *Tmem151a*^*−/*−^ mice in 48 h. **m** Duration of spontaneous dyskinesia attacks in *Tmem151a*^*−/−*^ mice. One dot represents one observed attack. **n** Representative images of dyskinesia attacks in a *Tmem151a*^−*/−*^ mouse.

To evaluate the frequency of identified *TMEM151A* variants in the general population, we performed Sanger sequencing in 1000 control individuals. Totally, we found four missense variants and three synonymous variants, which were predicted to be benign by bioinformatic software (Supplementary Fig. S1 and Table S4). None of the *TMEM151A* variants identified in PKD patients was found in controls. We then screened *TMEM151A* rare variants (MAF < 1%) in gnomAD and found 115 damaging missense variants, 6 truncated variants, and 1 in-frame deletion in 792 individuals. There was a significant enrichment of potentially pathogenic *TMEM151A* variants in PKD patients, compared with the gnomAD database (11/36 vs 792/~76,000, *P* = 8.05 × 10^−14^, Supplementary Table S5). In total, we identified 11 *TMEM151A* variants in 3 PKD pedigrees and 8 isolated patients, whose detailed clinical features are summarized in Supplementary Tables S6 and S7.

*TMEM151A* is a poorly characterized gene whose function is largely unknown. It is highly conserved among species (Supplementary Fig. S2). To explore its expression pattern, we measured the level of mice *Tmem151a* mRNA by real-time PCR. We found *Tmem151a* was highly expressed in the central nervous system (CNS), including the brain and spinal cord, followed by the stomach (Fig. 1c). *Tmem151a* was relatively low during the embryonic period, markedly increased during postnatal stages, peaked at postnatal day 14 (P14), and remarkably declined in adulthood (Fig. 1d). Real-time PCR and in situ hybridization analyses in P14 mice revealed that *Tmem151a* was ubiquitously expressed in the CNS, with a high level in the cerebral cortex, hippocampus, spinal cord, brainstem, and thalamus (Fig. 1e–g). On a more granular level, *Tmem151a* was enriched in cortical layers of the cerebral cortex and CA1 of the hippocampus (Fig. 1h). In the cerebellum, *Tmem151a* was mainly expressed in white matter, not in granule cells or Purkinje cell layers (Fig. 1h).

To examine the subcellular localization of Tmem151a protein, we generated EGFP-tagged wild-type (WT) *Tmem151a* plasmids and transfected them into COS-7 cells and cortical neurons. We found Tmem151a colocalized with endoplasmic reticulum (ER) marker Calnexin in COS-7 cells (Fig. 1i) and distributed in both axons and dendrites in primary cortical neurons (Fig. 1j). To elucidate the pathogenicity of *TMEM151A* non-truncated variants (p.L47P, p.48_51delLTLL, p.L253P, p.F288S, and p.S297T), we investigated the alternation of subcellular localization and protein expression of mutant TMEM151A. Cos-7 cells were transfected with WT and mutant EGFP-TMEM151A plasmids. Immunostaining revealed that mutant TMEM151A protein still retained on ER (data not shown). We then quantitatively measured the protein expression level of these *TMEM151A* variants by transfecting WT and mutant pIRES2-Flag-TMEM151A plasmids into HEK 293T cells. Western blot showed a significantly decreased protein expression of mutant TMEM151A compared to WT TMEM151A (Fig. 1k), suggesting a potential loss of function mechanism for these mutant TMEM151A.

We further generated *Tmem151a* knockout mice by CRISPR/Cas9-mediated genome editing according to our previous report^6^ and obtained *Tmem151a*^−/−^ and *Tmem151a*^+/−^ mice by breeding. Spontaneous dyskinesia was observed in both founder mice and F1 *Tmem151a*^−/−^ mice. Within 48 h, eight times of dyskinesia attacks were observed in four out of eight 1-month-old *Tmem151a*^−/−^ mice (Fig. 1l). No dyskinesia attacks were observed in *Tmem151a*^+/−^ mice and WT mice. The duration of episodes ranged from 10 to 37 s (Fig. 1m). After dyskinesia attacks (Fig. 1n and Supplementary Movie S1), the mice recovered to normal locomotion.

PKD is a hereditary disorder with autosomal dominant inheritance. However, a significant proportion of PKD patients seem to be sporadic^7^. Incomplete penetrance, de novo mutagenesis or autosomal recessive inheritance may account for this phenomenon. In this study, we found *TMEM151A* variants in three autosomal dominant families and eight isolated patients. Variants c.140 T > C, c.739 G > T, and c.623_624insA identified in three isolated patients were derived from one of their parents, who reported no obvious kinesigenic attacks. DNA samples were not available in the parents of the remaining five patients. We conjecture *TMEM151A* variants may have decreased penetrance, which has been observed in *PRRT2* and genes responsible for dystonia^8,9^. Besides, *TMEM151A* variants could also be de novo in isolated patients.

The potential mechanisms underlying PKD are not entirely clear. The identification of *PRRT2* as the first causative gene of PKD has improved our understanding of the pathogenesis of the disease. Recent studies indicate that PRRT2 acts on the presynaptic terminal and plays an important role in regulating synaptic transmission and neuronal excitability^10,11^. TMEM151A is predicted to be a transmembrane protein. We found TMEM151A was localized at ER in COS-7 cells. It is known that ER is the main intracellular Ca^2+^ store and plays a crucial role in intracellular Ca^2+^ mobilization and dynamics^12^. Considering the paroxysmal feature of PKD, we surmise TMEM151A may be an ER-associated Ca^2+^ channel. Alternatively, TMEM151A may interact with Ca^2+^ sensors and endow the SNARE complex, like the PRRT2 protein^13^. Given that patients with *TMEM151A* variants also obtain significant remission after carbamazepine treatment, it is possible that TMEM151A is an ion channel protein. Whether TMEM151A acts like PRRT2 in modulating Na^+^ channel is unclear^14^, which should be elucidated in the future. Loss of function might be the potential mechanism of mutant TMEM151A causing PKD. The decreased protein expression of non-truncated variants was in consistent with the mechanisms of haploinsufficiency. How amino acid residue changes affect TMEM151A protein functions requires further investigation.

In summary, we identified *TMEM151A* variants in both familial and isolated PKD patients, indicating that *TMEM151A* variants cause PKD. Although the function of TMEM151A remains elusive, we believe our findings will deepen the understanding of the mechanisms of PKD.

## Supplementary information

Supplementary information

Supplementary Movie S1

## Data Availability

The original data that support the findings are available from the corresponding author (Zhi-Ying Wu) on reasonable request.
