# Author Correction: *TMEM151A* variants cause paroxysmal kinesigenic dyskinesia

**DOI:** 10.1038/s41421-021-00345-3

**Published:** 2021-10-28

**Authors:** Hong-Fu Li, Yu-Lan Chen, Ling Zhuang, Dian-Fu Chen, Hua-Zhen Ke, Wen-Jiao Luo, Gong-Lu Liu, Sheng-Nan Wu, Wen-Hao Zhou, Zhi-Qi Xiong, Zhi-Ying Wu

**Affiliations:** 1 Department of Neurology and Research Center of Neurology in Second Affiliated Hospital, Zhejiang University School of Medicine, and Key Laboratory of Medical Neurobiology of Zhejiang Province, Hangzhou, Zhejiang, China; 2 Institute of Neuroscience and State Key Laboratory of Neuroscience, CAS Center for Excellence in Brain Science and Intelligence Technology, Chinese Academy of Sciences, Shanghai, China; 3 University of Chinese Academy of Sciences, Beijing, China; 4 Department of Neurology, Huashan Hospital, Shanghai Medical College, Fudan University, Shanghai, China; 5 Laboratory for Molecular Diagnostics, Shanghai Children’s Hospital, Shanghai Jiao Tong University, Shanghai, China; 6 Department of Neonatology, Children’s Hospital, Fudan University, Shanghai, China

**Keywords:** Endoplasmic reticulum, Rare variants

Correction to: *Cell Discovery* (2021)7:83

10.1038/s41421-021-00322-w Published online 13 September 2021

In the original publication of this “Correspondence”^1^, we made some mistakes in the annotation of genotypes in Fig. 1a.

The correct genotypes in Family 2 are as follows: II1 (+/+), II2 (NA), II3 (+/+), II4 (p.C125X), and II5 (+/+). In addition, a half round bracket was missed after “(p.S297T” in Fig. 1b. The correctly labelled Fig. 1a and b is displayed as below. This correction does not affect the description of the results or the conclusion of this work.
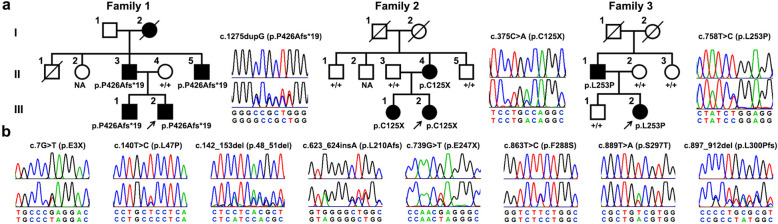


## References

[1] Li, H. et al. *TMEM151A* variants cause paroxysmal kinesigenic dyskinesia. *Cell Discov*. **7**, 83 (2021). 10.1038/s41421-021-00322-w PMC8437987 34518509

