# Supplementary material for: TMEM151A variants cause paroxysmal kinesigenic dyskinesia
Source: Cell Discov. 2021 Sep 13;7:83. doi: 10.1038/s41421-021-00322-w (PMC8437987; doi:10.1038/s41421-021-00322-w)
Supplement: Supplementary file 1 — Supplementary information [file 41421_2021_322_MOESM1_ESM.docx]

**Supplemental information**

**Methods and Materials**

**Subject and sample collection**

Thirty-six PKD probands without *PRRT2* variants and their available familial members were recruited at the Huashan Hospital of Fudan University between January 2011 and December 2014, and at the Second Affiliated Hospital of Zhejiang University School of Medicine between January 2015 and June 2020. All patients fulfilled the diagnosis criteria of PKD and had a favorable response to carbamazepine or oxcarbazepine by half-year follow-up. In addition, 1000 unrelated healthy individuals of matched ethnicity were included as control subjects. Genomic DNA of each participant was extracted from peripheral blood using QIAamp DNA Blood Mini Kits (QIAGEN, Germany). This study was approved by the Ethics Committee at each clinical site. All the participants or their guardians provided written informed consent.

**Whole exome sequencing**

Whole-exome sequencing (WES) was performed in 5 PKD families including 13 patients and 4 unaffected members, and 31 isolated PKD patients using an Agilent SureSelect Human All Exon (V6/V7) capture kit or Roche NimbleGen SeqCap EZ v3.0 (64M). The captured reads were sequenced on the Illumina NovaSeq 6000 platform or Illumina X-ten platform. We used the Burrows-Wheeler Aligner-MEM software package to align the reads to the human genome reference (hs37d5). Variants were called using the Genome Analysis Toolkit and annotated with ANNOVARA.

**Sanger sequencing**

Primers were designed to amplify the entire coding regions and corresponding splice junctions of *TMEM151A*. Polymerase chain reaction (PCR) amplification was carried out using LA Taq DNA polymerase with GC buffers (Takara). The detailed primers and annealing temperature for PCR are shown in Supplementary Table S8. Sanger sequencing was performed by an ABI 3700 or 3500xL automated sequencer (Applied Biosystems). Sequencing results were analyzed with Chromas software (Technelysium Pty Ltd) and Mutation Surveyor software (SoftGenetics, LLC).

**In situ hybridization**

P14 C57BL/6 mice (SLAC, Shanghai, China) were anesthetized, perfused with PBS and fixed with 4% paraformaldehyde (PFA) in PBS (pH = 7.4). The brain and spinal cord were removed and fixed with 4% PFA for another 16 hours, followed by consecutive dehydration in 15% and 30% gradient sucrose. Slices with a thickness of 16 μm were prepared and mounted on RNase-free superfrost glass slides (Fisher Scientific). Slices were subjected to ISH using the RNAscope 2.5 HD Regent Kit-BROWN (Advanced Cell Diagnostics, Cat. 300031) with the customized mouse Tmem151a C1 probe. All reagents were RNase-free, and instruments were DEPC-treated before use. Images were captured with a 20× objective lens on an Olympus VS120 microscope.

**Real-time PCR**

Tissues from C57BL/6 mice were homogenized on ice, and mRNA was extracted using TRIzol Reagent (Invitrogen). Eight hundred nanograms of mRNA per sample was reverse transcribed using the iScript cDNA Synthesis kit (Bio-Rad). Real-time PCR analysis of Tmem151a expression was performed using the SYBR Green I Master kit (Roche) in the LightCycler 480 Instrument (Roche). Tmem151a mRNA expression was normalized to the endogenous housekeeping gene GAPDH.

**Constructed plasmids**

PCR products of mouse Tmem151a from mouse brain cDNA were cloned into the pCAG-N1-EGFP vector digested with EcoR I and BamH I enzymes using a Seamless Cloning Kit (Beyotime). The human *TMEM151A* cDNA clone (NM_153266, Cat.CH804446) was purchased from Vigene Biosciences. The coding sequence of human *TMEM151A* and Flag-tag were cloned into the pIRES2-EGFP vector and the former was also cloned into the pEGFP-C2 vector for expression plasmids construct. Variants of *TMEM151A* were introduced into the expression plasmids by PCR-based site-directed mutagenesis or ClonExpress^®^ II One Step Cloning Kit (Vazyme) according to the manufacturer’s instruction. All mutant constructs were verified by sequencing. The primers used in this study were listed in Supplementary Table S9.

**Cell culture, transfecting and imaging**

COS-7 and HEK 293T cells purchased from the Shanghai Institute of Biochemistry and Cell Biology were cultured in DMEM supplemented with 10% fetal bovine serum (FBS). The cells were transfected with Lipofectamine 3000 (Invitrogen) according to the manufacturer’s instructions. Primary cortical neurons were prepared from postnatal 0 (P0) C57BL/6 mice and cultured in Neurobasal medium with 1% B27 and 10 mM GlutaMax. For in vitro electroporation, neurons were suspended in transfect medium (200 µL) mixed with pCAG-TMEM151A-EGFP plasmids. Then, neurons were transferred into a 2.0-mm electroporation cuvette (Fisher) and electroporated by an Amaxa Nucleofector apparatus. COS-7 cells and primary cortical neurons were fixed in 4% PFA-PBS supplemented with 4% sucrose 2 and 3 days after transfection, respectively. COS-7 cells and neurons were washed with PBS, blocked with 5% BSA plus 0.2% Triton X-100 for 2 h at room temperature, and then incubated with primary antibodies overnight. The next day, the cells were washed three times with PBS and then incubated with fluorescent secondary antibody plus DAPI for 2 h at room temperature. Then, cells were mounted onto glass slides. Images were acquired on a NIKON C2 microscope with a 60× oil objective and processed with ImageJ software. The antibodies were as follows: Calnexin (1:500, Abcam, ab10286), EGFP (1:1000, Abcam, ab13970) and Tau1 (1:1000, Millipore, MAB3420).

**Western Blot**

Transfection of HEK 293T cells with wild-type or mutant pIRES2-Flag-TMEM151A plasmids was performed using Lipofectamine 3000 (Invitrogen). Total protein was extracted after two days of transfection and then separated by 10% SDS-polyacrylamide gel electrophoresis (Bio-Rad) and blotted onto 0.45 µm PVDF membranes (Millipore). Membranes were blocked with 5% skim milk in TBS plus 0.1% Tween-20 for 2 h at room temperature and then incubated with primary antibodies overnight at 4 °C and the corresponding secondary antibody conjugated with HRP the next day. The following primary antibodies were used: anti Flag (1:2000, Sigma, F1804), anti GFP (1:5000, Santa Cruz, sc-9996), and HRP-conjugated GAPDH (1:10000, ABclonal, AC035). Bands were visualized by enhanced chemiluminescence (Thermo Scientific). Band intensities were measured with ImageJ software.

**Animals**

All animal procedures were approved by the Animal Care and Use Committee of the Institute of Neuroscience, Chinese Academy of Sciences. *Tmem151a* knockout mice in a C57BL/6J background were generated by CRISPR/Cas9-mediated genome editing, according to the previously described protocols. Briefly, five different single-guide RNAs (sgRNAs, guide RNA target site: sgRNA1, sgRNA2, sgRNA3, sgRNA4, sgRNA5) targeting the exon 1 and exon 2 of *Tmem151a* gene were used to generate insertion-deletion mutations (indels) which make protein translation stop prematurely. The sgRNAs and Cas9 mRNA were synthesized *in vitro* using MEGA shortscript T7 Transcription Kit (Life Technologies) and mMESSAGE mMACHINE T7 ULTRA Transcription Kit (Life Technologies). Subsequently, mixtures of sgRNAs and Cas9 mRNA were co-injected into C57BL/6J zygotes. Qualified embryos at blastocyst stage were implanted into surrogate mice. Tail genomic DNA of 50 founder mice was extracted, and PCR-amplified using a pair of primer targeting *Tmem151a* (Forward 5’-GCGGCTAGAGAAACCAGGCGTC-3’ and Reverse 5’-AGCACTTGGTG AAACGCAGCC-3’). The PCR products were purified with the FastPure Gel DNA Extraction Mini Kit (Vazyme Biotech Co., Ltd) and cloned into T-Vector. At least 20 clones for each mouse sample were sequenced for the analysis of *Tmem151a* mutation pattern. For generating of homozygous mice, we selected suitable founders for mating to obtain F1 mice. We also selected founders mating with wild-type mice to obtain heterozygous mice.

**Statistical analysis**

Statistical analyses were performed using SPSS 22 (IBM). The comparison of the frequency of *TMEM151A* variants between PKD patients and individuals in the gnomAD database was conducted using the Fisher’s exact test. To recognize differences in protein expression level, one-way ANOVA test and Dunnett’s multiple comparisons test were used to compare band intensities by GraphPad Prism software version 7.0. Variables were expressed as means and standard deviations of the mean. A *p*-value of less than 0.05 was considered statistically significant.

**Supplementary Figure**


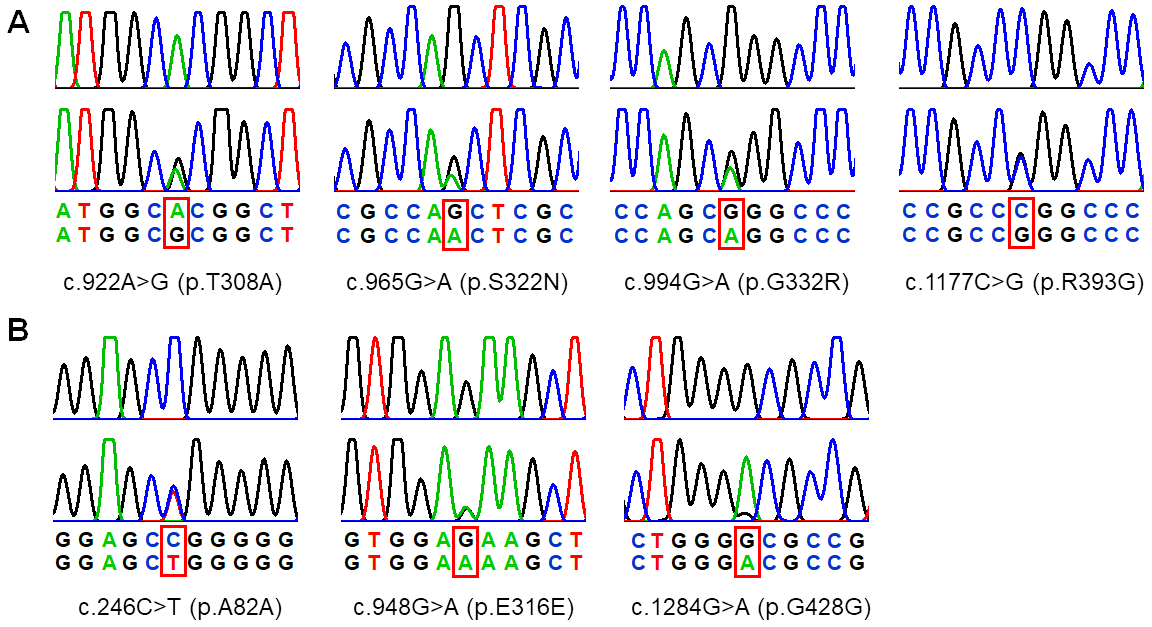


**Supplementary Fig. S1 Sequencing chromatograms of *TMEM151A* variants identified in 1000 Han Chinese control individuals**. There are 4 missense variants (A) and 3 synonymous variants (B). The upper chromatogram represents the normal sequence and the lower represents the mutant one.


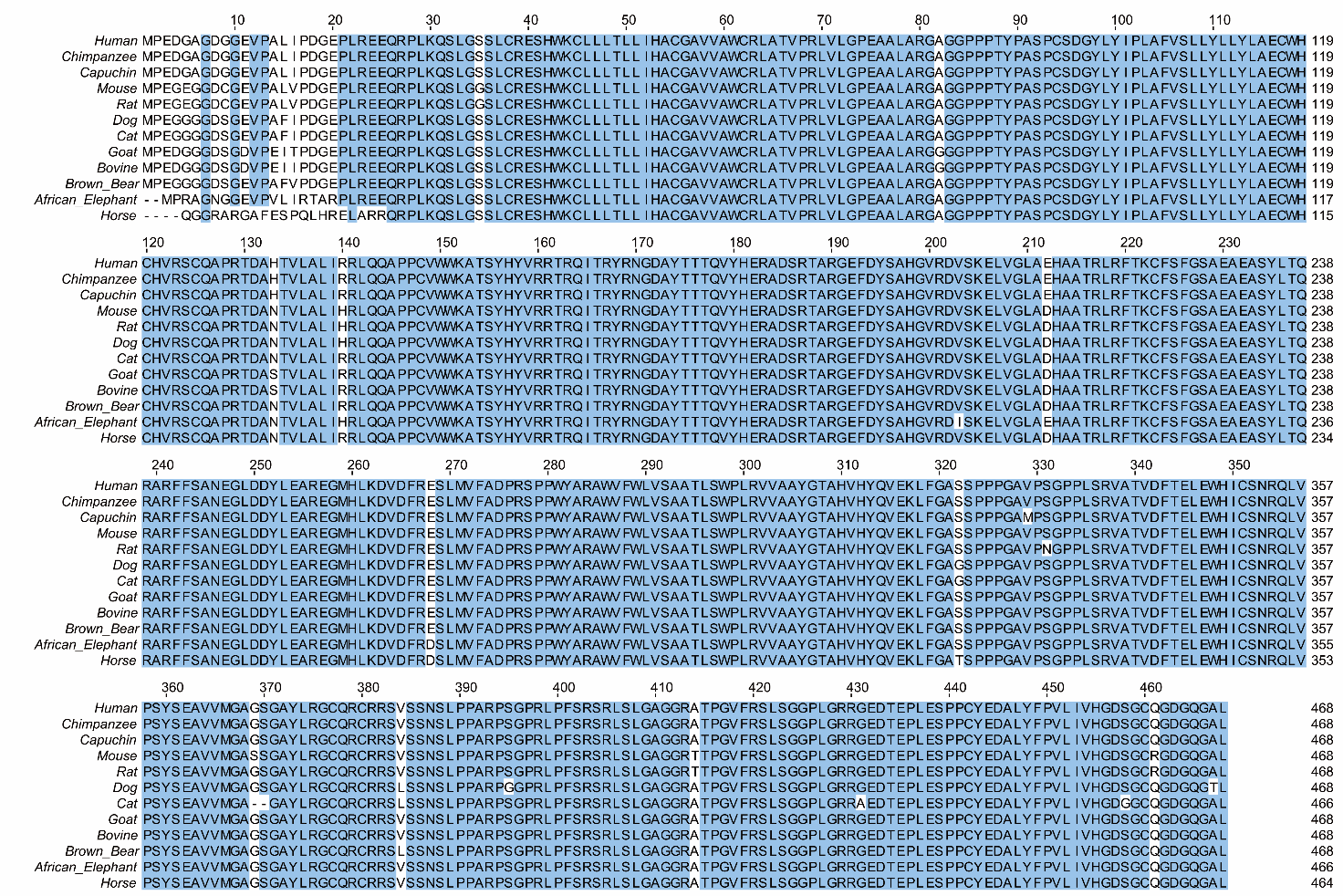


**Supplementary Fig. S2 Protein sequences alignment of TMEM151A homologs.** Protein sequences are obtained from the Uniprot database (Human Q8N4L1; Chimpanzee H2Q463; Capuchin A0A2K5SE06; Mouse Q6GQT5; Rat M0RAG0; Dog E2RGC1; Cat A0A337S908; Goat A0A452FBN4; Bovine A4IFG4; Brown Bear A0A3Q7TS80; African Elephant G3U220; Horse A0A3Q2GUU7) and aligned by Clustal Omega. Amino acids sharing ≥ 90% identity in all the species above are highlighted with blue. All residues of identified missense variants and the in-frame deletion were conserved in vertebrates. Edit, Jalview software.

**Supplementary Table S1.** Candidate genes identified in Family 1 to 3 using whole exome sequencing

|  | Gene | Refseq | Nucleotide change | Amino acid change |
| --- | --- | --- | --- | --- |
| Family 1 | *PAK4* | NM_001014831.2 | c.830C>G | p.T277S |
|  | *C12orf49* | NM_001353623.2 | c.8C>T | p.P3L |
|  | *OR5D18* | NM_001001952.1 | c.860A>C | p.N287T |
|  | *PUM1* | NM_001020658.1 | c.899G>C | p.G300A |
|  | *SRRM2* | NM_016333.3 | c.1694G>C | p.R565T |
|  | *MAP4K1* | NM_007181.4 | c.1658T>C | p.M553T |
|  | *FHL2* | NM_001039492.2 | c.629T>C | p.L210P |
|  | *LONRF2* | NM_198461.3 | c.58G>C | p.A20P |
|  | *C19orf53* | NM_014047.2 | c.244G>A | p.A82T |
|  | ***TMEM151A*** | NM_153266.3 | c.1275dupG | p.P426Afs*19 |
|  | *SPHK1* | NM_182965.2 | c.29delG | p.R10Pfs*51 |
| Family 2 | *LEFTY1* | NM_020997.3 | c.428_433del | p.143_145del |
|  | *ANKRD2* | NM_001346793.1 | c.545T>C | p.F182S |
|  | *LRRC10B* | NM_001145077.1 | c.672C>G | p.D224E |
|  | ***TMEM151A*** | NM_153266.3 | c.375C>A | p.C125X |
|  | *LEMD3* | NM_014319.4 | c.483C>G | p.D161E |
|  | *MSRB3* | NM_198080.3 | c.208G>A | p.A70T |
|  | *SLAIN1* | NM_001242868.1 | c.134A>T | p.Q45L |
|  | *SOX21* | NM_007084.3 | c.295G>A | p.G99S |
|  | *SOX1* | NM_005986.2 | c.1088C>T | p.A363V |
|  | *VIPAS39* | NM_001193314.1 | c.912+1G>T |  |
|  | *MAP2K1* | NM_002755.3 | c.86A>G | p.N29S |
|  | *PLXDC1* | NM_020405.4 | c.300C>A | p.S100R |
|  | *PLCD3* | NM_133373.5 | c.439C>A | p.R147S |
|  | *ZNF750* | NM_024702.2 | c.1687G>C | p.A563P |
|  | *MAN2B1* | NM_000528.3 | c.1016T>C | p.V339A |
|  | *FCGBP* | NM_003890.2 | c.2954C>T | p.A985V |
|  | *DACT3* | NM_145056.2 | c.1498C>G | p.P500A |
|  | *CADPS* | NM_003716.3 | c.2140G>A | p.E714K |
|  | *OR5H15* | NM_001005515.1 | c.161A>G | p.H54R |
|  | *HTT* | NM_002111.8 | c.2948T>C | p.I983T |
|  | *MAN2B2* | NM_015274.2 | c.196C>G | p.R66G |
|  | *BOD1L1* | NM_148894.2 | c.2307G>C | p.E769D |
|  | *PAICS* | NM_001079524.1 | c.1063C>T | p.P355S |
|  | *FAM46A* | NM_017633.2 | c.116_117insCGGCGACTTCGGCGG | p.G39delinsGGDFGG |
|  | *ROS1* | NM_002944.2 | c.152A>C | p.H51P |
|  | *MRPS33* | NM_053035.2 | c.23C>A | p.A8D |
|  | *MAPK15* | NM_139021.2 | c.487G>A | p.G163S |
|  | *TONSL* | NM_013432.4 | c.2246G>A | p.S749N |
|  | *NACC2* | NM_144653.4 | c.1591G>A | p.E531K |
| Family 3 | *TRNP1* | NM_001013642.2 | c.684A>G | p.X228W |
|  | *ZZZ3* | NM_015534.5 | c.1320A>G | p.I440M |
|  | *FAM72D* | NM_001345942.1 | c.68G>A | p.W23X |
|  | *CASQ1* | NM_001231.4 | c.1028C>A | p.A343D |
|  | *DNAH14* | NM_001373.1 | c.10496C>G | p.A3499G |
|  | *ITPKB* | NM_002221.3 | c.1990T>C | p.F664L |
|  | *PKDCC* | NM_138370.2 | c.8G>A | p.R3H |
|  | *GKN2* | NM_182536.2 | c.472C>T | p.H158Y |
|  | *DNAH6* | NM_001370.1 | c.1008G>C | p.K336N |
|  | *RAB6C* | NM_032144.2 | c.35G>A | p.R12K |
|  | *TTN* | NM_001267550.2 | c.104482G>C | p.E34828Q |
|  | *SESTD1* | NM_178123.4 | c.1708C>T | p.R570C |
|  | *OR6B3* | NM_173351.1 | c.929G>A | p.C310Y |
|  | *ITPR1* | NM_001168272.1 | c.775A>G | p.K259E |
|  | *ZDHHC3* | NM_016598.2 | c.619C>G | p.L207V |
|  | *RASA2* | NM_006506.3 | c.97G>C | p.E33Q |
|  | *SLC9B1* | NM_139173.3 | c.536A>G | p.H179R |
|  | *TIFA* | NM_052864.2 | c.281C>G | p.T94S |
|  | *TMEM232* | NM_001039763.3 | c.201G>T | p.L67F |
|  | *PPP2R2B* | NM_181675.3 | c.57_58insAGCAGCAGCAGCAGC | p.C20delinsSSSSSC |
|  | *FAM220A* | NM_001037163.1 | c.112C>G | p.P38A |
|  | *NACAD* | NM_001146334.1 | c.137C>T | p.T46I |
|  | *SUMF2* | NM_001130069.2 | c.662A>T | p.K221M |
|  | *SAMD9* | NM_017654.3 | c.1237T>G | p.Y413D |
|  | *RP1* | NM_006269.1 | c.350T>C | p.L117P |
|  | *GRINA* | NM_000837.1 | c.826C>G | p.R276G |
|  | *HMCN2* | NM_001291815.1 | c.5756C>T | p.S1919L |
|  | *HMCN2* | NM_001291815.2 | c.8284C>T | p.R2762W |
|  | *PHYH* | NM_006214.3 | c.935T>C | p.F312S |
|  | *PKP3* | NM_007183.3 | c.109G>T | p.E37X |
|  | ***TMEM151A*** | NM_153266.3 | c.758T>C | p.L253P |
|  | *C11orf80* | NM_024650.3 | c.74_75insGGGGGCGGCGGCGGC | p.E25delinsEGAAAA |
|  | *AIP* | NM_003977.3 | c.13A>G | p.I5V |
|  | *CEP295* | NM_033395.1 | c.604_607del | p.E203Qfs*45 |
|  | *DYNC2H1* | NM_001080463.1 | c.5155A>G | p.I1719V |
|  | *EMG1* | NM_006331 | c.455A>G | p.Q152R |
|  | *C1RL* | NM_016546.3 | c.425A>G | p.Q142R |
|  | *SLC17A8* | NM_139319.2 | c.102A>C | p.R34S |
|  | *ATXN3* | NM_004993.5 | c.915_916ins(CAG)_14_CA | p.G306fs |
|  | *AHNAK2* | NM_138420.3 | c.11273C>G | p.T3758R |
|  | *GOLGA8K* | NM_001282493.1 | c.1652C>T | p.P551L |
|  | *TARSL2* | NM_152334.2 | c.2377A>G | p.T793A |
|  | *TRAF7* | NM_032271.2 | c.235C>G | p.P79A |
|  | *MPRIP* | NM_015134.3 | c.537_539del | p.179_180del |
|  | *GAST* | NM_000805.4 | c.239T>C | p.L80P |
|  | *ANKRD12* | NM_015208.4 | c.682G>A | p.V228I |
|  | *RPS19* | NM_001022.3 | c.128A>G | p.K43R |
|  | *PTOV1* | NM_001305108.1 | c.33G>C | p.R11S |
|  | *LILRB4* | NM_001278430.3 | c.458T>C | p.F153S |
|  | *CCDC116* | NM_001331066.1 | c.1345C>T | p.L449F |
|  | *ZMAT5* | NM_019103.2 | c.11G>A | p.R4Q |
|  | *PLA2G6* | NM_003560.3 | c.1250C>A | p.P417H |

| **Supplementary Table S2.** Exome sequencing data of 5 PKD families and 31 isolated PKD patients | | | | | |
| --- | --- | --- | --- | --- | --- |
| Individual | Total sequence (reads) | Mapping rate (%) | Mean read depth | % of exome  >10x | % of exome  >20x |
| Roche NimbleGen SeqCap EZ v3.0 (64Mb), Illumina X-ten platform | | | | | |
| Isolated case F10 | 147,755,592 | 99.93 | 192 | 97.82 | 95.88 |
| Isolated case F23 | 108,442,718 | 99.96 | 141 | 97.48 | 94.82 |
| Isolated case F25 | 121,605,166 | 99.97 | 158 | 97.78 | 95.45 |
| Isolated case F26 | 122,517,522 | 99.94 | 160 | 97.53 | 95.07 |
| Isolated case F28 | 106,322,712 | 99.91 | 139 | 97.51 | 94.86 |
| Isolated case F29 | 125,347,700 | 99.94 | 164 | 97.65 | 95.28 |
| Isolated case F31 | 129,000,180 | 99.94 | 169 | 98.02 | 95.97 |
| Isolated case F33 | 125,162,224 | 99.95 | 163 | 97.72 | 95.44 |
| Isolated case F36 | 125,393,646 | 99.94 | 163 | 97.66 | 95.30 |
| Isolated case F54 | 143,644,046 | 99.95 | 185 | 97.01 | 94.44 |
| Isolated case F58 | 111,357,724 | 99.95 | 147 | 97.55 | 95.04 |
| Isolated case F60 | 129,208,548 | 99.94 | 170 | 97.69 | 95.39 |
| Isolated case F61 | 135,528,970 | 99.95 | 178 | 97.77 | 95.59 |
| Isolated case F62 | 129,407,156 | 99.96 | 170 | 97.88 | 95.73 |
| Isolated case F63 | 136,211,664 | 99.96 | 179 | 98.08 | 96.08 |
| Isolated case F72 | 146,911,056 | 99.96 | 193 | 98.25 | 96.41 |
| Isolated case F74 | 134,114,944 | 99.97 | 176 | 98.06 | 96.06 |
| Isolated case F75 | 159,566,182 | 99.97 | 210 | 98.44 | 96.82 |
| Isolated case F76 | 126,481,654 | 99.96 | 166 | 97.92 | 95.80 |
| Isolated case F85 | 123,912,296 | 99.95 | 153 | 98.06 | 96.19 |
| Isolated case F86 | 189,441,842 | 99.95 | 244 | 98.8 | 97.46 |
| Isolated case F94 | 128,051,790 | 99.97 | 164 | 97.84 | 95.53 |
| Agilent SureSelect Human All Exon (V6/V7), Illumina NovaSeq 6000 platform | | | | | |
| Individual | Total sequence (reads/bases) | Mapping rate (%) | Mean read depth | % of exome  >5x | % of exome  >20x |
| Family1 (II-3) | 90,159,946 | 99.53 | 193 | 99.54 | 98.33 |
| Family1 (II-4) | 103,127,546 | 99.71 | 222 | 99.45 | 98.60 |
| Family1 (II-5) | 53,727,026 | 99.71 | 115 | 99.55 | 96.60 |
| Family1 (III-1) | 100,295,794 | 99.73 | 213 | 99.63 | 98.63 |
| Family1 (III-2) | 87,838,394 | 99.83 | 117 | 99.57 | 95.26 |
| Family2 (II-3) | 58,077,600 | 99.48 | 122 | 99.48 | 96.79 |
| Family2 (II-4) | 48,519,416 | 99.60 | 101 | 99.29 | 95.69 |
| Family2 (III-1) | 59,180,086 | 99.65 | 124 | 99.41 | 97.26 |
| Family2 (III-2) | 78,048,956 | 99.88 | 108 | 99.33 | 94.45 |
| Family3 (II-1) | 64,789,648 | 99.61 | 137 | 99.63 | 97.91 |
| Family3 (II-2) | 70,702,578 | 99.76 | 158 | 99.45 | 97.92 |
| Family3 (III-2) | 67,430,102 | 99.66 | 146 | 99.41 | 97.65 |
| Family4 (19369) | 71,373,112 | 99.98 | 119 | 99.29 | 95.87 |
| Family4 (19369’s father) | 70,812,722 | 99.86 | 147 | 99.68 | 98.01 |
| Family5 (16284) | 66,386,750 | 99.98 | 116 | 99.30 | 93.90 |
| Family5 (16284’s father) | 76,621,112 | 99.88 | 165 | 99.70 | 97.94 |
| Family5 (16284’s mother) | 73,861,686 | 99.83 | 156 | 99.55 | 98.33 |
| Isolated case 19349 | 68,718,400 | 99.88 | 147 | 99.64 | 97.72 |
| Isolated case X18 | 89,567,674 | 99.94 | 133 | 99.41 | 96.83 |
| Isolated case X139 | 102,671,390 | 99.70 | 156 | 99.61 | 97.45 |
| Isolated case X372 | 107,439,194 | 99.31 | 153 | 99.71 | 97.56 |
| Isolated case X535 | 104,979,920 | 99.89 | 158 | 99.66 | 97.43 |
| Isolated case X536 | 100,544,038 | 99.90 | 149 | 99.66 | 97.30 |
| Isolated case X699 | 69,475,084 | 99.97 | 137 | 99.39 | 97.02 |
| Isolated case X847 | 10.94G (bases) | 99.87 | 123 | 99.74 | 97.83 |
| Isolated case X994 | 11.13G (bases) | 99.89 | 114 | 99.67 | 97.32 |

**Supplementary Table S3.** Features of *TMEM151A* variants identified in PKD patients

| Nucleotide  change | Amino acid  change | 1000G | ExAC | ESP6500 | gnomAD | ChinaMAP | Control | MutationTaster | SIFT | Polyphen-2 | CADD (score) |
| --- | --- | --- | --- | --- | --- | --- | --- | --- | --- | --- | --- |
| c.7G>T | p.E3X | 0 | 0 | 0 | 0 | 0 | 0 | Disease causing | NA | NA | Damaging (35) |
| c.140T>C | p.L47P | 0 | 8.41E-06 | 7.70E-05 | 8.28E-06 | 0 | 0 | Disease causing | Damaging | Probably damaging | Damaging (23.6) |
| c.142_153del | p.48_51del | 0 | 0 | 0 | 0 | 0 | 0 | Disease causing | NA | NA | NA |
| c.375C>A | p.C125X | 0 | 0 | 0 | 8.59E-06 | 4.72E-05 | 0 | Disease causing | NA | NA | Damaging (36) |
| c.623_624insA | p.L210Afs*136 | 0 | 0 | 0 | 0 | 0 | 0 | Disease causing | NA | NA | NA |
| c.739G>T | p.E247X | 0 | 0 | 0 | 0 | 0 | 0 | Disease causing | NA | NA | Damaging (37) |
| c.758T>C | p.L253P | 0 | 0 | 0 | 0 | 0 | 0 | Disease causing | Damaging | Probably damaging | Damaging (25.5) |
| c.863T>C | p.F288S | 0 | 0 | 0 | 0 | 0 | 0 | Disease causing | Damaging | Probably damaging | Damaging (25.3) |
| c.889T>A | p.S297T | 0 | 0 | 0 | 0 | 0 | 0 | Disease causing | Tolerable | Possibly damaging | Damaging (22.7) |
| c.897_912del | p.L300Pfs*118 | 0 | 0 | 0 | 0 | 0 | 0 | Disease causing | NA | NA | NA |
| c.1275dupG | p.P426Afs*19 | 0 | 0 | 0 | 0 | 0 | 0 | Disease causing | NA | NA | NA |

Abbreviations: 1000G = the 1000 Genomes Project; ExAC = Exome Aggregation Consortium; ESP6500 = Exome Sequencing Project v.6500; gnomAD = the Genome Aggregation Database; ChinaMAP = China Metabolic Analytics Project; Control = 1000 healthy controls in this study; SIFT = Sorting Intolerant From Tolerant; Polyphen-2 = Polymorphism Phenotyping v2; CADD = The Combined Annotation Dependent Depletion. NA = Not Available.

**Supplementary Table S4.** *TMEM151A* variants in 1000 ethnicity matched control individuals

| Nucleotide change | Amino acid change | Numbers | 1000G | ExAC | ESP6500 | gnomAD | ChinaMAP | Mutation Taster | SIFT | | Polyphen-2 | | CADD (score) | | |  |
| --- | --- | --- | --- | --- | --- | --- | --- | --- | --- | --- | --- | --- | --- | --- | --- | --- |
| c.922A>G | p.T308A | 1 | 0 | 0 | 0 | 0 | 0 | Disease causing | Damaging | | Possibly damaging | | Tolerable (5.345) | | |  |
| c.965G>A | p.S322N | 1 | 0 | 3.48E-05 | 0 | 1.23E-05 | 0 | Polymorphism | Tolerable | | Benign | | Tolerable (0.140) | | |  |
| c.994G>A | p.G332R | 7 | 0 | 0 | 0 | 5.85E-05 | 0.0009 | Disease causing | Tolerable | | Possibly damaging | | Tolerable (19.5) | | |  |
| c.1177C>G | p.R393G | 1 | 0 | 0 | 0 | 0 | 0 | Disease causing | Tolerable | | Probably damaging | | Tolerable (18.92) | | |  |
|  |  |  |  |  |  |  |  | HSF | | | | NatGene2 | | | NNSplice | |
| c.246C>T | p.A82A | 5 | 0 | 3.32E-05 | 0 | 4.14E-05 | 0.00095 | alteration of ESE/ESS ratio | | no alteration on DS/AS | | | | no alteration on DS/AS | |  |
| c.948G>A | p.E316E | 1 | 0 | 0 | 0 | 0 | 0 | alteration of ESE/ESS ratio | | no alteration on DS/AS | | | | no alteration on DS/AS | |  |
| c.1284G>A | p.G428G | 2 | 0 | 0.0002 | 0 | 3.56E-05 | 0.0008 | no significant impact | | no alteration on DS/AS | | | | no alteration on DS/AS | |  |

Abbreviations: NA = Not Available; HSF = Human Splicing Finder; ESE = Exonic Splicing Enhancer; ESS = Exonic Splicing Silencer; DS = Donor Site; AS = Acceptor Site.

**Supplementary Table S5.** List of rare *TMEM151A* truncated variants, missense variants and in-frame deletions with a minor allele frequency less than 1% present in gnomAD version 3.1, and the pathogenicity prediction by SIFT and CADD

| Chrom | Position | Variant | Protein | Type | Allele Count | Allele Number | SIFT | CADD |
| --- | --- | --- | --- | --- | --- | --- | --- | --- |
| 11 | 66292017 | c.4C>T | p.Pro2Ser | missense | 1 | 151936 | Damaging | 26.6 |
| 11 | 66292023 | c.10G>A | p.Asp4Asn | missense | 15 | 151966 | Tolerable | 14.98 |
| 11 | 66292033 | c.20G>A | p.Gly7Asp | missense | 174 | 152030 | Damaging | 25.7 |
| 11 | 66292053 | c.40G>A | p.Ala14Thr | missense | 1 | 152048 | Tolerable | 19.9 |
| 11 | 66292054 | c.41C>T | p.Ala14Val | missense | 1 | 151998 | Tolerable | 22.6 |
| 11 | 66292056 | c.43C>A | p.Leu15Ile | missense | 3 | 152004 | Tolerable | 16.16 |
| 11 | 66292063 | c.50C>G | p.Pro17Arg | missense | 2 | 151962 | Tolerable | 20.9 |
| 11 | 66292067 | c.54C>G | p.Asp18Glu | missense | 2 | 151924 | Tolerable | 22.9 |
| 11 | 66292068 | c.55G>A | p.Gly19Ser | missense | 1 | 151972 | Tolerable | 13.74 |
| 11 | 66292071 | c.58G>C | p.Glu20Gln | missense | 1 | 151944 | Tolerable | 18.07 |
| 11 | 66292075 | c.62C>A | p.Pro21Gln | missense | 1 | 151860 | Damaging | 23.2 |
| 11 | 66294325 | c.79C>T | p.Arg27Trp | missense | 1 | 152246 | Damaging | 32 |
| 11 | 66294326 | c.80G>T | p.Arg27Leu | missense | 2 | 152180 | Damaging | 28.2 |
| 11 | 66294328 | c.82C>T | p.Pro28Ser | missense | 7 | 152238 | Damaging | 25.9 |
| 11 | 66294332 | c.86T>C | p.Leu29Pro | missense | 1 | 152226 | Tolerable | 24.6 |
| 11 | 66294344 | c.98T>C | p.Leu33Pro | missense | 1 | 152210 | Damaging | 25.2 |
| 11 | 66294361 | c.115C>T | p.Arg39Cys | missense | 1 | 152230 | Damaging | 33 |
| 11 | 66294386 | c.140T>C | p.Leu47Pro | missense | 3 | 152216 | Damaging | 23.6 |
| 11 | 66294391 | c.145A>G | p.Thr49Ala | missense | 1 | 152124 | Damaging | 23.6 |
| 11 | 66294418 | c.172G>A | p.Val58Met | missense | 2 | 152262 | Damaging | 24.5 |
| 11 | 66294429 | c.183G>C | p.Trp61Cys | missense | 1 | 152242 | Damaging | 26.8 |
| 11 | 66294430 | c.184T>C | p.Cys62Arg | missense | 1 | 152254 | Damaging | 24.5 |
| 11 | 66294440 | c.194C>T | p.Ala65Val | missense | 33 | 152248 | Damaging | 19.02 |
| 11 | 66294442 | c.196A>G | p.Thr66Ala | missense | 1 | 152210 | Tolerable | 5.757 |
| 11 | 66294473 | c.227C>A | p.Ala76Asp | missense | 1 | 152232 | Tolerable | 11.06 |
| 11 | 66294475 | c.229G>A | p.Ala77Thr | missense | 181 | 152206 | Tolerable | 1.292 |
| 11 | 66294482 | c.236C>A | p.Ala79Asp | missense | 20 | 152212 | Tolerable | 5.852 |
| 11 | 66294484 | c.238C>T | p.Arg80Trp | missense | 1 | 152162 | Tolerable | 13.41 |
| 11 | 66294485 | c.239G>A | p.Arg80Gln | missense | 1 | 152174 | Tolerable | 1.18 |
| 11 | 66294487 | c.241G>A | p.Gly81Arg | missense | 1 | 152204 | Damaging | 22.9 |
| 11 | 66294493 | c.247G>A | p.Gly83Arg | missense | 4 | 152188 | Tolerable | 2.611 |
| 11 | 66294500 | c.254C>T | p.Pro85Leu | missense | 1 | 152210 | Tolerable | 1.481 |
| 11 | 66294500 | c.254C>A | p.Pro85Gln | missense | 4 | 152210 | Tolerable | 0.036 |
| 11 | 66294508 | c.262A>G | p.Thr88Ala | missense | 2 | 152068 | Tolerable | 6.601 |
| 11 | 66294514 | c.268C>T | p.Pro90Ser | missense | 1 | 152228 | Tolerable | 7.229 |
| 11 | 66294522 | c.276C>G | p.Ser92Arg | missense | 1 | 152234 | Damaging | 23.4 |
| 11 | 66294545 | c.299A>G | p.Tyr100Cys | missense | 1 | 152192 | Damaging | 23.4 |
| 11 | 66294551 | c.305C>G | p.Pro102Arg | missense | 3 | 152204 | Damaging | 25.2 |
| 11 | 66294562 | c.316G>A | p.Val106Ile | missense | 2 | 152170 | Tolerable | 16.51 |
| 11 | 66294565 | c.319T>G | p.Ser107Ala | missense | 1 | 152150 | Tolerable | 7.09 |
| 11 | 66294580 | c.334C>A | p.Leu112Ile | missense | 1 | 152238 | Tolerable | 20.9 |
| 11 | 66294598 | c.352T>C | p.Trp118Arg | missense | 1 | 152230 | Damaging | 24.9 |
| 11 | 66294601 | c.355C>T | p.His119Tyr | missense | 1 | 152230 | Damaging | 23.9 |
| 11 | 66294608 | c.362A>G | p.His121Arg | missense | 2 | 152132 | Tolerable | 6.676 |
| 11 | 66294610 | c.364G>A | p.Val122Met | missense | 2 | 152206 | Tolerable | 15.45 |
| 11 | 66294610 | c.364G>C | p.Val122Leu | missense | 22 | 152206 | Tolerable | 14.14 |
| 11 | 66294611 | c.365T>C | p.Val122Ala | missense | 1 | 152220 | Tolerable | 1.144 |
| 11 | 66294614 | c.368G>A | p.Arg123Gln | missense | 1 | 152150 | Damaging | 23.8 |
| 11 | 66294631 | c.385C>A | p.Arg129Ser | missense | 1 | 152166 | Damaging | 27.3 |
| 11 | 66294631 | c.385C>G | p.Arg129Gly | missense | 1 | 152166 | Damaging | 26.2 |
| 11 | 66294638 | c.392A>G | p.Asp131Gly | missense | 1 | 152182 | Damaging | 22.9 |
| 11 | 66294639 | c.393C>A | p.Asp131Glu | missense | 1 | 152154 | Tolerable | 11.03 |
| 11 | 66294643 | c.397C>G | p.His133Asp | missense | 5 | 152136 | Tolerable | 0.003 |
| 11 | 66294647 | c.401C>A | p.Thr134Lys | missense | 1 | 152242 | Damaging | 23.9 |
| 11 | 66294656 | c.410C>T | p.Ala137Val | missense | 2 | 152238 | Tolerable | 22.3 |
| 11 | 66294656 | c.410C>A | p.Ala137Glu | missense | 11 | 152238 | Tolerable | 10.91 |
| 11 | 66294665 | c.419G>A | p.Arg140His | missense | 24 | 152248 | Tolerable | 20.8 |
| 11 | 66294667 | c.421C>T | p.Arg141Trp | missense | 1 | 152260 | Damaging | 26.4 |
| 11 | 66294668 | c.422G>A | p.Arg141Gln | missense | 1 | 152242 | Tolerable | 23.7 |
| 11 | 66294673 | c.427C>G | p.Gln143Glu | missense | 2 | 152254 | Damaging | 24 |
| 11 | 66294674 | c.428A>G | p.Gln143Arg | missense | 2 | 152248 | Tolerable | 18.88 |
| 11 | 66294680 | c.434C>A | p.Ala145Glu | missense | 1 | 152256 | Damaging | 29.4 |
| 11 | 66294719 | c.473A>G | p.Tyr158Cys | missense | 1 | 152220 | Damaging | 24.3 |
| 11 | 66294727 | c.481C>T | p.Arg161Cys | missense | 1 | 152242 | Damaging | 28.7 |
| 11 | 66294728 | c.482G>A | p.Arg161His | missense | 5 | 152228 | Damaging | 24.8 |
| 11 | 66294731 | c.485C>A | p.Thr162Lys | missense | 1 | 152252 | Tolerable | 23.4 |
| 11 | 66294760 | c.514G>A | p.Asp172Asn | missense | 2 | 152234 | Tolerable | 28 |
| 11 | 66294785 | c.539A>G | p.Tyr180Cys | missense | 1 | 152240 | Damaging | 24.1 |
| 11 | 66294790 | c.544G>A | p.Glu182Lys | missense | 1 | 152234 | Damaging | 25.9 |
| 11 | 66294796 | c.550G>A | p.Ala184Thr | missense | 1 | 152230 | Damaging | 22.8 |
| 11 | 66294797 | c.551C>G | p.Ala184Gly | missense | 1 | 152254 | Damaging | 19.07 |
| 11 | 66294806 | c.560G>T | p.Arg187Leu | missense | 4 | 152252 | Damaging | 23 |
| 11 | 66294811 | c.565G>T | p.Ala189Ser | missense | 1 | 152246 | Tolerable | 10.16 |
| 11 | 66294817 | c.571G>C | p.Gly191Arg | missense | 1 | 152208 | Damaging | 18.86 |
| 11 | 66294833 | c.587C>T | p.Ser196Leu | missense | 1 | 152240 | Damaging | 20.5 |
| 11 | 66294835 | c.589G>C | p.Ala197Pro | missense | 18 | 152224 | Tolerable | 19.78 |
| 11 | 66294836 | c.590C>A | p.Ala197Glu | missense | 1 | 152220 | Tolerable | 22.8 |
| 11 | 66294844 | c.598G>A | p.Val200Ile | missense | 1 | 152238 | Damaging | 25.6 |
| 11 | 66294854 | c.608T>G | p.Val203Gly | missense | 1 | 152168 | Damaging | 24.8 |
| 11 | 66294857 | c.611C>T | p.Ser204Phe | missense | 1 | 152226 | Damaging | 25.8 |
| 11 | 66294862 | c.616G>A | p.Glu206Lys | missense | 1 | 152222 | Tolerable | 21.9 |
| 11 | 66294864 | c.618G>T | p.Glu206Asp | missense | 1 | 152246 | Tolerable | 21.4 |
| 11 | 66294872 | c.626G>A | p.Gly209Glu | missense | 1 | 152230 | Tolerable | 22.7 |
| 11 | 66294877 | c.631G>A | p.Ala211Thr | missense | 1 | 152238 | Tolerable | 13.58 |
| 11 | 66294878 | c.632C>T | p.Ala211Val | missense | 1 | 152206 | Damaging | 22.6 |
| 11 | 66294885 | c.639C>A | p.His213Gln | missense | 3 | 152220 | Tolerable | 22.5 |
| 11 | 66294887 | c.641C>T | p.Ala214Val | missense | 1 | 152234 | Damaging | 23.6 |
| 11 | 66294895 | c.649C>T | p.Arg217Trp | missense | 1 | 152242 | Damaging | 26.4 |
| 11 | 66294896 | c.650G>A | p.Arg217Gln | missense | 1 | 152198 | Damaging | 27.7 |
| 11 | 66294902 | c.656G>T | p.Arg219Leu | missense | 1 | 152226 | Damaging | 28.8 |
| 11 | 66294910 | c.664A>G | p.Lys222Glu | missense | 1 | 152148 | Damaging | 25.4 |
| 11 | 66294913 | c.667T>A | p.Cys223Ser | missense | 2 | 152178 | Tolerable | 22.3 |
| 11 | 66294925 | c.679G>C | p.Gly227Arg | missense | 2 | 152204 | Damaging | 26.8 |
| 11 | 66294931 | c.685G>T | p.Ala229Ser | missense | 1 | 152230 | Damaging | 15.02 |
| 11 | 66294937 | c.691G>T | p.Ala231Ser | missense | 1 | 152208 | Tolerable | 16.41 |
| 11 | 66294956 | c.710C>T | p.Thr237Met | missense | 1 | 152200 | Damaging | 24 |
| 11 | 66294962 | c.716G>A | p.Arg239Gln | missense | 2 | 152212 | Damaging | 28.4 |
| 11 | 66294965 | c.719C>A | p.Ala240Glu | missense | 1 | 152182 | Tolerable | 25.8 |
| 11 | 66294967 | c.721C>T | p.Arg241Cys | missense | 1 | 152192 | Damaging | 29.9 |
| 11 | 66294968 | c.722G>A | p.Arg241His | missense | 7 | 152190 | Tolerable | 28.5 |
| 11 | 66294983 | c.737A>G | p.Asn246Ser | missense | 8 | 152178 | Damaging | 22.9 |
| 11 | 66294987 | c.741G>T | p.Glu247Asp | missense | 1 | 152202 | Damaging | 24.6 |
| 11 | 66294997 | c.751G>A | p.Asp251Asn | missense | 2 | 152186 | Damaging | 29.3 |
| 11 | 66294998 | c.752A>G | p.Asp251Gly | missense | 3 | 152118 | Damaging | 26.1 |
| 11 | 66295015 | c.769G>A | p.Glu257Lys | missense | 1 | 152174 | Damaging | 27.4 |
| 11 | 66295025 | c.779A>T | p.His260Leu | missense | 36 | 152094 | Tolerable | 23.4 |
| 11 | 66295037 | c.791T>C | p.Val264Ala | missense | 1 | 152146 | Damaging | 23.5 |
| 11 | 66295040 | c.794A>C | p.Asp265Ala | missense | 1 | 152140 | Damaging | 24.1 |
| 11 | 66295046 | c.800G>A | p.Arg267His | missense | 1 | 152190 | Damaging | 25.5 |
| 11 | 66295054 | c.808C>T | p.Leu270Phe | missense | 3 | 152168 | Damaging | 24.4 |
| 11 | 66295057 | c.811A>G | p.Met271Val | missense | 1 | 152090 | Tolerable | 0.003 |
| 11 | 66295057 | c.811A>C | p.Met271Leu | missense | 13 | 152090 | Tolerable | 0.04 |
| 11 | 66295058 | c.812T>C | p.Met271Thr | missense | 1 | 152066 | Tolerable | 11.8 |
| 11 | 66295059 | c.813G>T | p.Met271Ile | missense | 1 | 152166 | Tolerable | 4.042 |
| 11 | 66295066 | c.820G>A | p.Ala274Thr | missense | 18 | 152172 | Tolerable | 15.61 |
| 11 | 66295073 | c.827C>T | p.Pro276Leu | missense | 4 | 152132 | Damaging | 25.7 |
| 11 | 66295073 | c.827C>G | p.Pro276Arg | missense | 1 | 152132 | Damaging | 24.4 |
| 11 | 66295075 | c.829C>T | p.Arg277Cys | missense | 317 | 152178 | Tolerable | 24.1 |
| 11 | 66295076 | c.830G>C | p.Arg277Pro | missense | 1 | 152156 | Tolerable | 9.256 |
| 11 | 66295091 | c.845A>G | p.Tyr282Cys | missense | 1 | 152052 | Damaging | 23.4 |
| 11 | 66295100 | c.854C>T | p.Ala285Val | missense | 2 | 152162 | Tolerable | 14.19 |
| 11 | 66295104 | c.858G>T | p.Trp286Cys | missense | 1 | 151912 | Tolerable | 13.92 |
| 11 | 66295105 | c.859G>A | p.Val287Ile | missense | 1 | 151836 | Tolerable | 9.459 |
| 11 | 66295129 | c.883A>T | p.Thr295Ser | missense | 1 | 151870 | Tolerable | 9.782 |
| 11 | 66295130 | c.884C>G | p.Thr295Arg | missense | 1 | 152030 | Damaging | 22.9 |
| 11 | 66295136 | c.890C>T | p.Ser297Leu | missense | 1 | 151998 | Damaging | 24.7 |
| 11 | 66295147 | c.901C>A | p.Arg301Ser | missense | 1 | 152038 | Damaging | 25 |
| 11 | 66295148 | c.902G>A | p.Arg301His | missense | 2 | 152040 | Damaging | 23.8 |
| 11 | 66295186 | c.940C>G | p.Gln314Glu | missense | 1 | 152086 | Tolerable | 19.38 |
| 11 | 66295205 | c.959G>A | p.Gly320Asp | missense | 1 | 152062 | Damaging | 25.6 |
| 11 | 66295211 | c.965G>A | p.Ser322Asn | missense | 1 | 152072 | Tolerable | 0.14 |
| 11 | 66295214 | c.968C>T | p.Ser323Leu | missense | 1 | 152060 | Tolerable | 11.47 |
| 11 | 66295216 | c.970C>T | p.Pro324Ser | missense | 1 | 152076 | Tolerable | 0.147 |
| 11 | 66295219 | c.973C>T | p.Pro325Ser | missense | 2 | 152096 | Tolerable | 0.002 |
| 11 | 66295223 | c.977C>G | p.Pro326Arg | missense | 1 | 152076 | Tolerable | 0.079 |
| 11 | 66295223 | c.977C>A | p.Pro326Gln | missense | 1 | 152076 | Tolerable | 1.112 |
| 11 | 66295226 | c.980G>A | p.Gly327Glu | missense | 1 | 152086 | Tolerable | 0.799 |
| 11 | 66295226 | c.980G>T | p.Gly327Val | missense | 1 | 152086 | Tolerable | 13.13 |
| 11 | 66295228 | c.982G>A | p.Ala328Thr | missense | 1 | 152036 | Tolerable | 2.745 |
| 11 | 66295228 | c.982G>T | p.Ala328Ser | missense | 1 | 152036 | Tolerable | 0.045 |
| 11 | 66295231 | c.985G>A | p.Val329Met | missense | 1 | 151996 | Tolerable | 0.501 |
| 11 | 66295231 | c.985G>T | p.Val329Leu | missense | 2 | 151996 | Tolerable | 0.055 |
| 11 | 66295240 | c.994G>A | p.Gly332Arg | missense | 3 | 152032 | Tolerable | 19.5 |
| 11 | 66295243 | c.997C>G | p.Pro333Ala | missense | 2 | 152064 | Tolerable | 1.154 |
| 11 | 66295243 | c.997C>T | p.Pro333Ser | missense | 1 | 152064 | Tolerable | 9.686 |
| 11 | 66295243 | c.997C>A | p.Pro333Thr | missense | 1 | 152064 | Tolerable | 9.959 |
| 11 | 66295247 | c.1001C>T | p.Pro334Leu | missense | 1 | 151882 | Tolerable | 19.42 |
| 11 | 66295249 | c.1003C>A | p.Leu335Met | missense | 1 | 152092 | Tolerable | 18.01 |
| 11 | 66295258 | c.1012G>A | p.Val338Met | missense | 2 | 151898 | Damaging | 26.1 |
| 11 | 66295262 | c.1016C>T | p.Ala339Val | missense | 1 | 152002 | Tolerable | 24.9 |
| 11 | 66295262 | c.1016C>A | p.Ala339Asp | missense | 1 | 152002 | Tolerable | 15.77 |
| 11 | 66295267 | c.1021G>A | p.Val341Met | missense | 1 | 152020 | Tolerable | 22.7 |
| 11 | 66295272 | c.1026C>G | p.Asp342Glu | missense | 3 | 152078 | Damaging | 26.2 |
| 11 | 66295291 | c.1045C>T | p.His349Tyr | missense | 1 | 152084 | Tolerable | 23.2 |
| 11 | 66295328 | c.1082C>G | p.Ser361Trp | missense | 1 | 152054 | Damaging | 28.8 |
| 11 | 66295340 | c.1094T>C | p.Val365Ala | missense | 1 | 151914 | Damaging | 21.2 |
| 11 | 66295343 | c.1097T>C | p.Met366Thr | missense | 1 | 151974 | Damaging | 23.9 |
| 11 | 66295345 | c.1099G>A | p.Gly367Ser | missense | 6 | 152036 | Tolerable | 23 |
| 11 | 66295349 | c.1103C>A | p.Ala368Glu | missense | 15 | 152028 | Tolerable | 17.5 |
| 11 | 66295349 | c.1103C>T | p.Ala368Val | missense | 1 | 152028 | Tolerable | 14.2 |
| 11 | 66295358 | c.1112G>A | p.Gly371Asp | missense | 2 | 152032 | Damaging | 9.226 |
| 11 | 66295371 | c.1125A>T | p.Arg375Ser | missense | 1 | 151614 | Damaging | 0.054 |
| 11 | 66295373 | c.1127G>A | p.Gly376Asp | missense | 6 | 151830 | Tolerable | 11.21 |
| 11 | 66295377 | c.1131C>G | p.Cys377Trp | missense | 1 | 151950 | Damaging | 23.1 |
| 11 | 66295381 | c.1135C>T | p.Arg379Cys | missense | 6 | 151814 | Damaging | 31 |
| 11 | 66295390 | c.1144C>T | p.Arg382Cys | missense | 3 | 151952 | Damaging | 26 |
| 11 | 66295403 | c.1157G>A | p.Ser386Asn | missense | 1 | 151902 | Tolerable | 11.2 |
| 11 | 66295409 | c.1163C>G | p.Ser388Trp | missense | 1 | 151854 | Tolerable | 24.5 |
| 11 | 66295415 | c.1169C>G | p.Pro390Arg | missense | 1 | 151900 | Tolerable | 18.51 |
| 11 | 66295417 | c.1171C>T | p.Pro391Ser | missense | 242 | 151748 | Tolerable | 5.457 |
| 11 | 66295418 | c.1172C>T | p.Pro391Leu | missense | 1 | 151776 | Tolerable | 15.17 |
| 11 | 66295420 | c.1174G>A | p.Ala392Thr | missense | 2 | 151796 | Tolerable | 11 |
| 11 | 66295423 | c.1177C>G | p.Arg393Gly | missense | 1 | 151722 | Tolerable | 18.92 |
| 11 | 66295423 | c.1177C>T | p.Arg393Trp | missense | 48 | 151722 | Damaging | 32 |
| 11 | 66295436 | c.1190C>G | p.Pro397Arg | missense | 1 | 152014 | Damaging | 20.5 |
| 11 | 66295449 | c.1203C>A | p.Phe401Leu | missense | 1 | 152020 | Tolerable | 4.219 |
| 11 | 66295453 | c.1207C>G | p.Arg403Gly | missense | 1 | 152016 | Tolerable | 15.25 |
| 11 | 66295453 | c.1207C>T | p.Arg403Cys | missense | 2 | 152016 | Tolerable | 23 |
| 11 | 66295460 | c.1214G>C | p.Arg405Pro | missense | 1 | 151990 | Damaging | 24.3 |
| 11 | 66295466 | c.1220C>T | p.Ser407Leu | missense | 1 | 152014 | Damaging | 22.8 |
| 11 | 66295471 | c.1225G>A | p.Gly409Ser | missense | 1 | 152026 | Tolerable | 13.47 |
| 11 | 66295474 | c.1228G>A | p.Ala410Thr | missense | 1 | 152000 | Tolerable | 9.892 |
| 11 | 66295493 | c.1247C>T | p.Pro416Leu | missense | 1 | 152086 | Tolerable | 7.016 |
| 11 | 66295498 | c.1252G>T | p.Val418Phe | missense | 1 | 152102 | Tolerable | 12.86 |
| 11 | 66295498 | c.1252G>A | p.Val418Ile | missense | 1 | 152102 | Tolerable | 12.81 |
| 11 | 66295499 | c.1253T>C | p.Val418Ala | missense | 64 | 151638 | Tolerable | 7.911 |
| 11 | 66295504 | c.1258C>T | p.Arg420Cys | missense | 3 | 152064 | Damaging | 32 |
| 11 | 66295508 | c.1262G>A | p.Ser421Asn | missense | 1 | 152066 | Damaging | 24.5 |
| 11 | 66295516 | c.1270G>C | p.Gly424Arg | missense | 1 | 152082 | Damaging | 23.6 |
| 11 | 66295519 | c.1273G>A | p.Gly425Arg | missense | 8 | 152124 | Damaging | 16.1 |
| 11 | 66295520 | c.1274G>A | p.Gly425Glu | missense | 1 | 152018 | Damaging | 8.767 |
| 11 | 66295523 | c.1277C>A | p.Pro426Gln | missense | 1 | 152022 | Tolerable | 10.05 |
| 11 | 66295528 | c.1282G>A | p.Gly428Arg | missense | 1 | 152014 | Tolerable | 12.03 |
| 11 | 66295532 | c.1286G>A | p.Arg429His | missense | 5 | 152004 | Damaging | 19.93 |
| 11 | 66295534 | c.1288C>T | p.Arg430Cys | missense | 2 | 151946 | Tolerable | 22.9 |
| 11 | 66295543 | c.1297G>A | p.Asp433Asn | missense | 1 | 152136 | Tolerable | 9.989 |
| 11 | 66295546 | c.1300A>C | p.Thr434Pro | missense | 1 | 151770 | Tolerable | 3.93 |
| 11 | 66295546 | c.1300A>G | p.Thr434Ala | missense | 1 | 151770 | Tolerable | 0.031 |
| 11 | 66295547 | c.1301C>T | p.Thr434Met | missense | 1 | 151984 | Damaging | 23.1 |
| 11 | 66295560 | c.1314G>T | p.Glu438Asp | missense | 1 | 151768 | Tolerable | 6.777 |
| 11 | 66295568 | c.1322C>T | p.Pro441Leu | missense | 1 | 152190 | Damaging | 27.1 |
| 11 | 66295574 | c.1328A>T | p.Tyr443Phe | missense | 1 | 152100 | Damaging | 24.2 |
| 11 | 66295603 | c.1357A>G | p.Ile453Val | missense | 1 | 152116 | Damaging | 23.6 |
| 11 | 66295607 | c.1361T>A | p.Val454Asp | missense | 2 | 152138 | Damaging | 26 |
| 11 | 66295607 | c.1361T>C | p.Val454Ala | missense | 2 | 152138 | Damaging | 21.7 |
| 11 | 66295611 | c.1365C>A | p.His455Gln | missense | 2 | 152160 | Damaging | 23.8 |
| 11 | 66295612 | c.1366G>C | p.Gly456Arg | missense | 1 | 152140 | Tolerable | 16.4 |
| 11 | 66295633 | c.1387G>A | p.Asp463Asn | missense | 1 | 151122 | Tolerable | 11.37 |
| 11 | 66295633 | c.1387G>T | p.Asp463Tyr | missense | 1 | 151122 | Tolerable | 23.9 |
| 11 | 66295641 | c.1395G>C | p.Gln465His | missense | 1 | 148846 | Tolerable | 7.686 |
| 11 | 66295646 | c.1400C>T | p.Ala467Val | missense | 1 | 145410 | Tolerable | 14.87 |
| 11 | 66294325 | c.81del | p.Leu29Ter | truncated | 1 | 152246 | NA | NA |
| 11 | 66294599 | c.353G>A | p.Trp118Ter | truncated | 1 | 152224 | NA | NA |
| 11 | 66294695 | c.449G>A | p.Trp150Ter | truncated | 1 | 152244 | NA | NA |
| 11 | 66295070 | c.829dup | p.Arg277ProfsTer69 | truncated | 1 | 151758 | NA | NA |
| 11 | 66295112 | c.866G>A | p.Trp289Ter | truncated | 3 | 151918 | NA | NA |
| 11 | 66295627 | c.1382del | p.Gln461ArgfsTer27 | truncated | 1 | 151716 | NA | NA |
| 11 | 66294566 | c.333_341del | p.Leu112_Leu114del | in-frame deletion | 2 | 152220 | NA | NA |

Note: *TMEM151A* transcript ID: ENST00000327259.5 / NM_153266.4; the prediction result of SIFT and CADD was obtained via the VarCards website^1^ (http://varcards.biols.ac.cn/). Missense variants with a prediction score more than 20 by CADD were considered to be damaging.

1. Li, J. *et al.* VarCards: an integrated genetic and clinical database for coding variants in the human genome. *Nucleic Acids Res*. **46**, D1039-D1048 (2018).

**Supplementary Table S6.** Clinical features of the patients with *TMEM151A* variants

| Patient/gender | Variant | At onset  (year) | At present  (year) | Duration (second) | Phenotype | Laterality | Treatment | Response  to drug |
| --- | --- | --- | --- | --- | --- | --- | --- | --- |
| Family 1 (II-3)/M | p.P426Afs*19 | 15 | 58 | 1-2 | D | Unilateral | No treatment | NA |
| Family 1 (II-5)/M | p.P426Afs*19 | 10 | 55 | 2-3 | D/E | Unilateral | PHT 100mg tid | Incomplete |
| Family 1 (III-1)/M | p.P426Afs*19 | 12 | 31 | 5-10 | D | Bilateral | CBZ 200mg qod | Incomplete |
| Family 1 (III-2)/M | p.P426Afs*19 | 9 | 27 | 5-10 | D | Bilateral | CBZ 200mg qd | Incomplete |
| Family 2 (II-4)/F | p.C125X | 10 | 52 | 5-10 | D | Bilateral | No treatment | NA |
| Family 2 (III-1)/F | p.C125X | 11 | 27 | 5-10 | D | Unilateral | No treatment | NA |
| Family 2 (III-2)/F | p.C125X | 11 | 25 | 5-10 | D/E | Bilateral | CBZ 50mg qd | Complete |
| Family 3 (II-1)/M | p.L253P | 10 | 52 | 5-10 | D | Unilateral | No treatment | NA |
| Family 3 (III-2)/F | p.L253P | 10 | 27 | 5-10 | D | Unilateral | CBZ 100mg qd | Incomplete |
| Isolated case 1/F | p.E3X | 9 | 18 | <10 | D/C | Bilateral | CBZ 100mg qd | Incomplete |
| Isolated case 2/F | p.L47P | 9 | 21 | 5-10 | D/C | Bilateral | CBZ 100mg bid | Complete |
| Isolated case 3/M | p.48_51del | 12 | 25 | 5-10 | D | Bilateral | CBZ 100mg qod | Incomplete |
| Isolated case 4/M | p.L210Afs*136 | 14 | 19 | 3-4 | D | Unilateral | CBZ 50mg qd | Complete |
| Isolated case 5/M | p.E247X | 12 | 22 | <10 | D | Bilateral | OXC 75mg bid | Incomplete |
| Isolated case 6/M | p.F288S | 13 | 34 | 5-6 | C | Bilateral | CBZ 50mg tid | Complete |
| Isolated case 7/M | p.S297T | 11 | 30 | <10 | D | Bilateral | CBZ 50mg bid | Incomplete |
| Isolated case 8/M | p.L300Pfs*118 | 12 | 32 | <10 | D | Bilateral | CBZ 100mg qd | Complete |

Abbreviations: F = female, M = male, D = dystonia, C = choreoathetosis, E = epileptiform attacks, CBZ = carbamazepine, OXC = oxcarbazepine, PHT = phenytoin, NA = Not Available.

| **Supplementary Table S7**. Detailed clinical features of PKD patients with *TMEM151A* variants | | | | | | | | | | | | |
| --- | --- | --- | --- | --- | --- | --- | --- | --- | --- | --- | --- | --- |
| Patient | Age (y)  /sex | AAO (y) | Remission age (y) | Trigger of attacks | Duration of  attacks (s) | Frequency  of attacks | Description  of attacks | Aura | Interictal EEG | Brain MRI | Comorbidity | Response to treatment |
| Family 1  (II-3) | 58/M | 15 | 30 | SM | 1-2 | hard to recall,  ~1/1-2 day(s) | no loss of consciousness, stiffness and twisting of left limbs. | Yes | NA | NA | No | No treatment |
| Family 1  (II-5) | 55/M | 10 | 26 | SM | 2-3 | hard to recall,  ~1/3-5 days | no loss of consciousness, dystonic movements of the right arm | No | NA | NA | Seizures (with loss of consciousness at 10 years old, screaming, limbs twitching, foaming at mouth, lasting about 10 minutes) | Good control on PHT, 100mg, Tid  (occasional attacks and attacks remitted at 26 years old) |
| Family 1  (III-1) | 31/M | 12 | No | SM | 5-10 | ~1/2-3 days | no loss of consciousness, twisting of the right arm and the left leg | Yes | NA | NA | No | Good control on CBZ, 200mg, Qod (occasional attacks and attacks recurred when he forgot to take the drug) |
| Family 1  (III-2) | 27/M | 9 | No | SM | 5-10 | up to  10-20/day | no loss of consciousness, stiffness and twisting of limbs, no attacks at sleep. | Yes | No | Normal | No | Good control on CBZ, 200mg, QD  (occasional attacks, and sometime a sensation of imminent attacks which didn’t happen in fact) |
| Family 2  (II-4) | 52/F | 10 | 23 | SM | 5-10 | hard to recall,  high frequency  at teenage | no loss of consciousness, dystonic movements，and could fall to the ground due to the weakness of lower limbs. | Yes | NA | NA | No | No treatment |
| Family 2  (III-1) | 27/F | 11 | No | SM | 5-10 | ~1/2-3 days | no loss of consciousness, twisting of the left limbs | Yes | NA | NA | No | No treatment  (occasional attacks) |
| Family 2  (III-2) | 25/F | 11 | No | SM | 5-10 | 4-6/day | no loss of consciousness, twisting of legs, feeling weakness | Yes | Normal | Normal | 1.Suspected seizures  (occasional episodes at sleep with loss of consciousness during the elementary school, no treatment) 2. Anxiety disorder | Reduced attacks on OXC; Complete control on CBZ, 50mg, QD. (attacks recurred when she forgot to take the drug) |
| Family 3  (II-1) | 52/M | 10 | 18 | SM, S | 5-10 | hard to recall | no loss of consciousness, twisting and stiffness of the left limbs | NA | NA | NA | No | No treatment |
| Family 3  (III-2) | 27/F | 10 | No | SM | 5-10 | 5-10/day | no loss of consciousness, twisting and stiffness of limbs, clawing of hands and grimacing; unilateral but alternating sides. | Yes | Normal | Normal | No | Good control on CBZ, 100mg, QD (occasional attacks, and sometime a sensation of imminent attacks which didn’t happen in fact) |
| Isolated case 1 | 18/F | 9 | No | SM, S, F | <10 | up to  10-30/day | no loss of consciousness, dystonic and choreic movements of limbs, grimacing, and sometimes the trunk lost balance and fell backward | Yes | No | No | No | Good control on CBZ, 100mg, QD (occasional attacks) |
| Isolated case 2 | 21/F | 9 | 20 | SM | 5-10 | no record | no loss of consciousness, dystonic and choreic movements of limbs | NA | Normal | Normal | No | Complete control on CBZ, 100mg, Bid |
| Isolated case 3 | 25/M | 12 | No | SM | 5-10 | 2-3/day | no loss of consciousness, twisting and stiffness of limbs, and grimacing | Yes | Normal | Normal | No | Good control on CBZ, 100mg Qod (occasional attacks, and sometime a sensation of imminent attacks which didn’t happen in fact) |
| Isolated case 4 | 19/M | 14 | No | SM | 3-4 | up to  >20/day | no loss of consciousness, twisting and stiffness of left limbs, dystonic postures, couldn’t walk, grimacing | Yes | Normal | Normal | No | Complete control on CBZ, 50mg, QD |
| Isolated case 5 | 22/M | 12 | No | SM, S | <10 | 5-6/day | no loss of consciousness, stiffness of upper limbs, grimacing, torticollis | Yes | Normal | Normal | No | Good control on OXC, 75mg, Bid (occasional attacks when he was startled and attacks recurred when he forgot to take the drug) |
| Isolated case 6 | 34/M | 13 | 27 | SM | 5-6 | 1-5/day | no loss of consciousness, choreic movements of limbs, tight teeth | Yes | NA | NA | No | Complete control on CBZ, 50mg Tid. |
| Isolated case 7 | 30/M | 11 | No | SM | <10 | hard to recall | no loss of consciousness, stiffness of limbs, dystonic postures, couldn’t walk, grimacing | Yes | Normal | Normal | No | Good control on CBZ, 50mg, Bid (occasional attacks and a sensation of imminent attacks which didn’t happen) |
| Isolated case 8 | 32/M | 12 | No | SM | <10 | up to 10-20/day | no loss of consciousness, stiffness of limbs, dystonic postures, the trunk could lean forward, mouth open, couldn’t speak, torticollis | Yes | Normal | Normal | No | Complete control on CBZ, 100mg qd. |

Abbreviations: F = female, M = male, AAO = age at onset, SM = sudden movement, S = stress/startle, NA = Not Available, CBZ = carbamazepine, OXC = oxcarbazepine, PHT = phenytoin, VPA = Valproate

**Supplementary Table S8.** PCR primers and conditions designed for *TMEM151A* gene

| Exon number |  | Oligonucleotide primers (5’→3’) | Size of PCR  product (bp) | Annealing temperature (°C) |
| --- | --- | --- | --- | --- |
| Exon 1 | F | 5’- GGTTAGACCAGCTCAAGGTCG -3’ | 524 | 60 |
|  | R | 5’- CTCTGGGACTTCCCTCCTCA -3’ |  |  |
| Exon 2.1 | F | 5’- GAGCTGGAAATGTGAAGCCTG -3’ | 572 | 62 |
|  | R | 5’- GCACGTAGTGATAGCTGGTGG -3’ |  |  |
| Exon 2.2 | F | 5-’ CCGATGGCTACCTGTACATCC -3’ | 511 | 62 |
|  | R | 5’- GTCTACGTCCTTCAGGTGCATG -3’ |  |  |
| Exon 2.3 | F | 5’- TGCGCTTCACCAAGTGCTTC -3’ | 514 | 62 |
|  | R | 5’- AGCGAGTTGCTGCTGACAGAG -3’ |  |  |
| Exon 2.4 | F | 5’- GTGGACTTCACTGAGCTCGAG -3’ | 566 | 60 |
|  | R | 5’- CCAAAATGACCACAGGTAACTGC -3’ |  |  |
| Exon 2.5 | F | 5’- CACCATTCCACCATGGGCTTAG -3’ | 573 | 62 |
|  | R | 5’- AGGTTGTAGGGGTAGCTGCATG -3’ |  |  |
| Exon 2.6 | F | 5’- CTGCTACTCACCCAACATTCC -3’ | 634 | 62 |
|  | R | 5’- ACTGGAGCCCAATTCTGAGTC -3’ |  |  |

**Supplementary Table S9.** Mutagenesis primers for mutant TMEM151A

| Mutation | Oligonucleotide primers (5’→3’)* | |
| --- | --- | --- |
| c.140T>C | F: | 5’- CACTGGAAGTGCCTGC**C**CCTCACGCTGCTCATC -3’ |
| (p.L47P) | R: | 5’- GATGAGCAGCGTGAGG**G**GCAGGCACTTCCAGTG -3’ |
| c.142_153del | F: | 5’- AAGTGCCTGCTCATCCACGCCTGCGGGGCCGTGGTGG -3’ |
| (p.48_51delLTLL) | R: | 5’- CGTGGATGAGCAGGCACTTCCAGTGCGACTCGCGGCA -3’ |
| c.758T>C | F: | 5’- CCTGGACGACTATC**C**GGAGGCGCGCGAGG -3’ |
| (p.L253P) | R: | 5’- CCTCGCGCGCCTCC**G**GATAGTCGTCCAGG -3’ |
| c.863T>C | F: | 5’- GCGCGCCTGGGTCT**C**CTGGCTCGTGTCGG -3’ |
| (p.F288S) | R: | 5’- CCGACACGAGCCAG**G**AGACCCAGGCGCGC -3’ |
| c.889T>A | F: | 5’- TCGGCGGCCACGCTG**A**CGTGGCCCCTGCG -3’ |
| (p.S297T) | R: | 5’- CGCAGGGGCCACG**T**CAGCGTGGCCGCCGA -3’ |

*Sequences of primers used to engineer the mutant constructs from the wild-type TMEM151A pIRES2-EGFP vector (Flag-tagged) and the pEGFP-C2 vector. The sites of the mutations were shown in bold.

**Supplementary Movie S1.**

Spontaneous dyskinesia attacks in one *Tmem151a*-deficient mice (*Tmem151a*^-/-^).
